# How common are taste and smell abnormalities in COVID-19? A systematic review and meta-analysis

**DOI:** 10.1016/j.jtumed.2021.10.009

**Published:** 2021-11-15

**Authors:** Shahzaib Ahmad, Anum Sohail, Muhammad Abubakar Shahid Chishti, Muhammad Aemaz Ur Rehman, Hareem Farooq

**Affiliations:** a Department of Medicine, King Edward Medical University, Lahore, Pakistan; b Department of Medicine, Mayo Hospital, King Edward Medical University, Lahore, Pakistan

**Keywords:** الشم, الذوق, كوفيد -19, اضطرابات الذوق, اضطرابات الشم, التحليل التلوي, COVID-19, Gustatory dysfunction, Meta-analysis, Olfaction disorders, Smell, Taste

## Abstract

**Objective:**

Olfactory and gustatory dysfunction (OGD) are important early clinical symptoms of COVID-19. We aim to calculate the pooled prevalence of these symptoms and discuss the likely implications on clinical practice such as their use as screening tools and potential prognosis indicators.

**Methods:**

Using a combination of keywords and medical subject headings, we searched for observational studies in the following five databases: Medline/PubMed, Scopus, Cochrane Library, Web of Science, and Google Scholar. Two authors independently screened and selected the final articles according to the inclusion criteria. Two investigators independently assessed the risk of bias in individual studies using the Newcastle–Ottawa Scale. Heterogeneity and publication bias were also assessed. The reported outcome of the pooled analysis was the prevalence of OGD calculated using a random-effect model. Preferred Reporting Items for Systematic Reviews and Meta-Analyses guidelines were followed to report results.

**Results:**

Seventeen studies with a total sample size of 4149 were included in this meta-analysis. Out of these, 2106 and 2676 patients reported some degree of olfactory and/or gustatory dysfunction with COVID-19, respectively. The reported outcomes were in terms of pooled prevalence, with gustatory dysfunction being 57.33% and olfactory dysfunction being 59.69%, a significantly high occurrence.

**Conclusion:**

There is a high occurrence of smell and taste impairment in COVID-19. Given the lack of objective testing for detecting OGD in most studies, the high prevalence found is likely to be an underestimation of the true prevalence. This implies that physicians must use them as reliable early indicators of COVID-19 and employ them before using expensive tests.

## الملخص

**أهداف البحث:**

يعد خلل الشم والذوق من الأعراض السريرية المبكرة الهامة لمرض كوفيد-19. تهدف الدراسة إلى حساب الانتشار التجميعي لهذه الأعراض؛ ثم مناقشة الآثار المحتملة على الممارسة السريرية مثل استخدامها كأدوات فحص ومؤشرات تشخيص محتملة.

**طرق البحث:**

باستخدام مجموعة من الكلمات المفتاحية ومصطلحات الميدلاين المعيارية، بحثنا عن الدراسات بالملاحظة في قواعد البيانات الخمس التالية: "ميدلاين" و "سكوبس" و"مكتبة كوكرين" و "شبكة العلوم" و " قوقل العلمي". قام اثنان من الباحثين بفحص واختيار الدراسات، كما تم تقييم احتمالات التحيز في الدراسات المضمنة باستخدام مقياس نيوكاسل-أوتاوا. تم اتباع إرشادات "بريزما" لفحص المقالات النهائية وتحديدها وفقا لمعايير التضمين. كانت النتيجة المبلغ عنها للتحليل التجميعي هي معدل انتشار ضعف الشم والذوق المحسوب باستخدام نموذج التأثير العشوائي.

**النتائج:**

تم تضمين 17 دراسة مع حجم عينة إجمالي 4149 في هذا التحليل التلوي. من بين هؤلاء، أبلغ 2106 و 2676 مريضا عن درجة من الخلل الوظيفي الشمي و/أو الذوقي مع كوفيد-19؛ على التوالي. كانت النتائج المبلغ عنها من حيث الانتشار التجميعي، مع ضعف الذوق 57.33٪ والخلل الشمي 59.69٪، والذي يعتبر انتشارا كبيرا.

**الاستنتاجات:**

هناك معدل مرتفع لحدوث ضعف في حاسة الشم والذوق في مرض كوفيد-19. نظرًا لعدم وجود اختبار موضوعي للكشف عن ضعف الشم والذوق في معظم الدراسات، فمن المرجح أن يكون معدل الانتشار المرتفع الذي اكتشفناه أقل من الانتشار الحقيقي. هذا يعني أنه يجب على الأطباء استخدامها كمؤشرات مبكرة موثوقة لمرض كوفيد-19 وتوظيفها قبل فحص المرضى باختبارات باهظة الثمن.

## Introduction

In December 2019, a few cases of a strange pneumonia-like disease were reported for the first time in Wuhan, China. On January 30, 2020, the alarming spread of this contagious disease, called novel coronavirus disease (COVID-19), led the World Health Organization (WHO) to declare a public health emergency of international concern (PHEIC).^1^^,^^2^ The common set of symptoms identified during the initial days of the pandemic were dry cough, fever, flu, shortness of breath, fatigue, and diarrhoea.^3^, ^4^, ^5^, ^6^ In addition to these, many confirmed cases of COVID-19 started presenting in hospitals with neurological manifestations such as smell and taste dysfunction.^7^, ^8^, ^9^ This dysfunction ranged from a decrease in taste (hypogeusia) and smell (hyposmia) to a complete loss of these sensations, commonly called ageusia and anosmia, respectively. In light of emerging evidence, the Centers for Disease Control and Prevention (CDC) eventually added ‘new loss of taste or smell’ to the list of symptoms associated with SARS-CoV-2 infection.

The interaction of SARS-CoV-2 with various receptors is proposed to play a role in its ability to cause widespread systemic manifestations. High neural and glial cell expression of angiotensin-converting enzyme (ACE-2) receptors, coupled with their abundance on nasal and tongue mucosa, provides reliable evidence to attribute OGD to SARS-CoV-2. Since other presenting symptoms such as fever, cough, and flu are non-specific, OGD can serve as a distinctive symptom to distinguish cases of COVID-19 from other respiratory infections with greater reliability.^8^^,^^10^, ^11^, ^12^

Numerous observational studies discussing the prevalence and extent of OGD have appeared in the literature from the virus's inception to date.^10^, ^11^, ^12^ The large-scale data available in the literature merit the use of a pooled qualitative and quantitative approach (systematic review and meta-analysis) to better understand these symptoms and report their numbers. This will not only allow physicians to objectively recognise the prevalence of OGD in COVID-19 but also set a foundation for future researchers to conduct a larger meta-analysis as the existing literature expands with time. Furthermore, a heavy financial burden, time constraints, and workforce utility are associated with diagnostic tests such as reverse transcription polymerase chain reaction (RT-PCR). This meta-analysis will serve to determine whether the prevalence of OGD is sufficiently high in COVID-19 to justify their use as screening tools for this disease. Some researchers have also suggested that OGD may be associated with prognosis in COVID-19 patients, but there is a significant disparity in their views. A concurrent review of the literature will also shed light on this aspect as a secondary objective.

## Materials and Methods

We conducted our systematic review and meta-analysis in keeping with the Preferred Reporting Items for Systematic Reviews and Meta-Analyses Protocol (PRISMA-P). All included studies were searched systematically. Our search identified a total of 25 studies, but only 17 could be included in the meta-analysis because of a difference in the reported outcome measures; our study used weighted summary proportion (pooled prevalence) as the main outcome measure. Hence, only descriptive data from the remaining eight studies are included.

### Data sources and search strategy

We searched five databases, namely PubMed, Scopus, Cochrane Library, Web of Science, and Google Scholar, for our study. Our search strategy included a combination of keywords and medical subject headings (MeSH), including some of the following: ‘covid∗’, OR ‘corona∗’, OR ‘Wuhan coronavirus’, OR ‘COVID-19’, OR ‘novel coronavirus’, OR ‘2019-nCoV’, OR ‘coronavirus’, OR ‘SARS-CoV-2’, OR ‘SARS-2’ OR ‘severe acute respiratory syndrome coronavirus 2’ AND ‘olfactory dysfunction’, OR ‘gustatory dysfunction’, OR ‘Anosmia’, OR ‘Ageusia’. The complete search strategy for PubMed is provided in Supplementary Files.

### Study selection and data extraction

**Figure 1:**
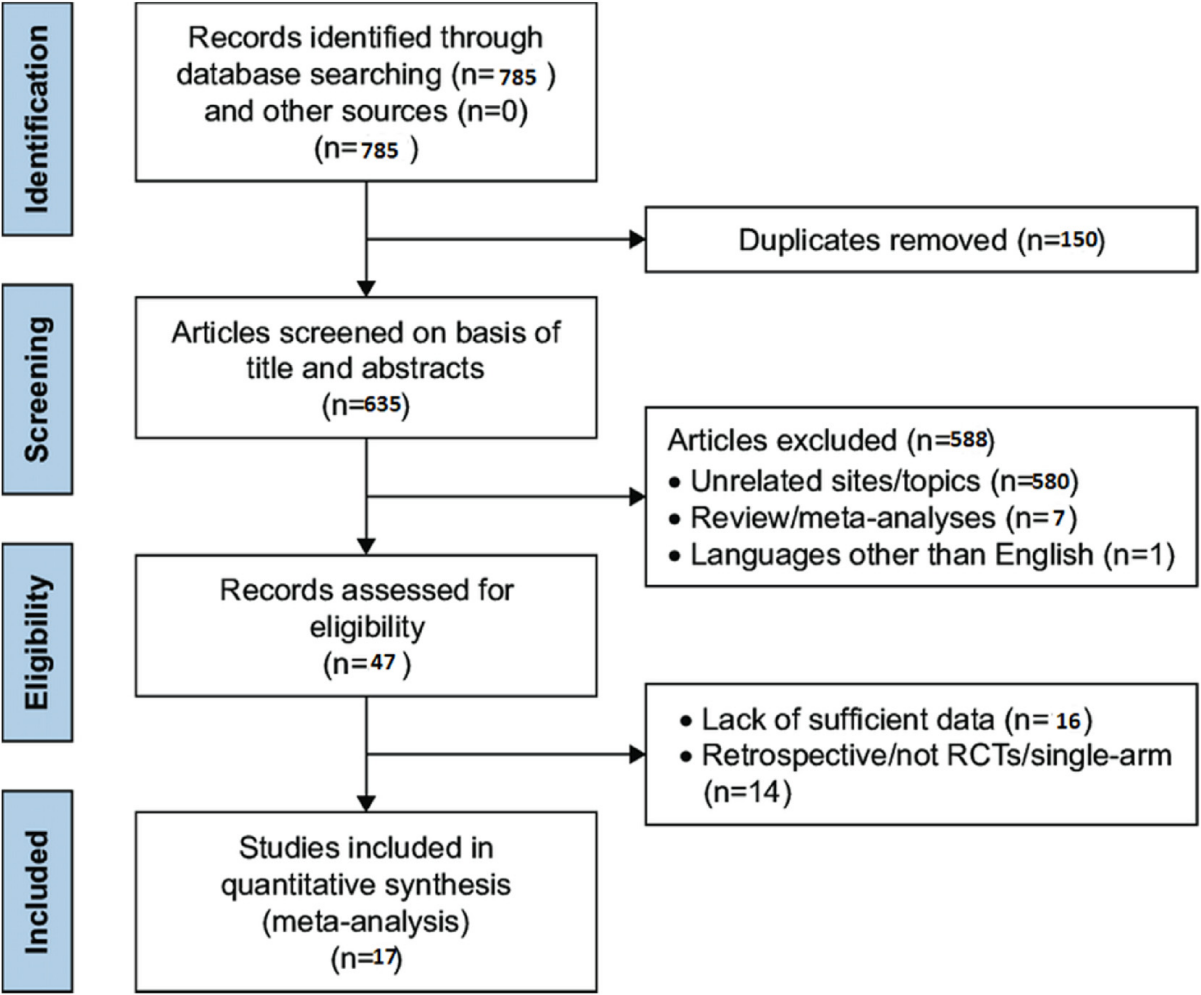
PRISMA flow diagram showing inclusion of studies.

Two authors (A. S. and S. A.) independently evaluated the search results. After removing duplicates, they identified the studies on the basis of the inclusion and exclusion criteria, and help was sought from a third author (MASC) if any confusion regarding inclusion arose. The authors of the articles were not contacted about the papers’ verifiability because these articles had already been published in peer-reviewed journals. Unreviewed materials were excluded from the analysis; therefore, the authors felt no need to contact the investigators. For the systematic review, the following data were searched, extracted, and analysed: name of the study, publication month and year, sample size, study design, COVID-19-associated OGD symptoms and their prevalence, and the presence of any other neurological clinical features. Literature and records were managed using Endnote X9 software. The study selection process is illustrated in PRISMA (Figure 1).

**Table 1:** Study and patient characteristics of included studies.

ID	Article	Study design	Publication date	Number of participants	Major olfactory symptom	Major gustatory symptoms	Olfactory symptoms N (%)	Gustatory symptoms N (%)	Any neurological manifestation
1	Hopkins C et al.^13^	Cohort study	April 7, 2020	2428	Anosmia	Hypogeusia	74.4% complete loss, 17.3% severe loss	90% reduced taste, but 61% could still differentiate among varied tastes	None
2	Luers et al.^14^	Cross-sectional study	April 6, 2020	72	Hyposmia	Hypogeusia	73.6%	69.4%	Headache
3	Paderno et al.^15^	Cohort study	June 15, 2020	151	Anosmia or Hyposmia	Hypogeusia	83%	89%	None
4	Petrocelli et al.^16^	Cohort study	July 28, 2020	300	Anosmia	Ageusia	70% (47% anosmia)	70% (38% ageusia)	None
5	Gorzkowski et al.^17^	Cross-sectional Study	June 29, 2020	229	Anosmia or Hyposmia	–	70.3%		None
6	Ceron et al.^18^	A prospective multicentric cohort study	July 3, 2020	55	Anosmia	Dysgeusia	51 (92.7%)	80%	None
7	Cho et al.^19^	Prospective cross-sectional study cohort		143	Anosmia	Dysgeusia	47%	43.4%	None
8	Rojas-lechuga et al.^20^	Cross-sectional study	July 31, 2020	197 107 control	–	–	70.1%	65%	None
9	Gomez-Iglesias et al.^21^	Online observational study	May 29, 2020	909	Anosmia, hyposmia	Ageusia, dysgeusia	824 (90.65%)	824 (90.65%)	None
10	Altin F et al.^22^	Prospective study	June 17, 2020	81	Anosmia, hyposmia	Ageusia, dysgeusia	50 (61.7%)	22 (27.2%)	None
11	Chary E et al.^23^	Multicentre case series study		115	Anosmia, hyposmia	Ageusia, dysgeusia	81 (70%)	81 (70%)	None
12	Chiesa estomba et al.^24^	Clinical study	April 28, 2020	542			449 (81.9%)	67.5% partial loss, 14.4% complete loss	Headache 72.5%
13	Qui et al.^12^	Multicentre case series study	May 15, 2020	394	Anosmia, hyposmia	Ageusia, dysgeusia	161 (41%)	161 (41%)	None
14	Meini et al.^25^	Phone review	May 29, 2020	100	Anosmia, hyposmia	Ageusia, dysgeusia	29	41	Headache
15	Carignan et al.^26^	Case-control study	May 27, 2020	2883	Anosmia	Dysgeusia	51.5%	63.4%	Blurred vision
16	Lechien et al.^27^	Objective evaluation	May 6, 2020	86	Anosmia, hyposmia		61.4% 41 anosmia, 12 hyposmia		Headache 60%
17	Speth et al.^28^	Prospective, cross-sectional	May 19, 2020	103	Anosmia, hyposmia, nasal obstruction, rhinorrhoea	Decrease and no sense of taste	14.6% hyposmia 46.6% anosmia	25.2% decreased sense of smell, 39.8% no sense of smell	None
18	Paderno et al.^29^	Cross-sectional study	May 11, 2020	508	Anosmia	Ageusia	(325) 64%	60%	Headache and syncope
19	Vaira et al.^30^	Observational study	April 14, 2020	72	Hyposmia/anosmia, rhinorrhoea, nasal obstruction	Hypogeusia, ageusia	14.4% isolated olfactory disorders	12.5% isolated taste disorders	Headache
20	Yan et al.^31^	Retrospective review	April 21, 2020	128	Hyposmia/anosmia, rhinorrhoea, sinusitis	Dysgeusia	Anosmia/hyposmia (26.9%) in admitted patients	Dysgeusia (23.1%) in admitted patients	Headache
21	Lechien et al.^32^	Multicentre study	April 6, 2020	417	Hyposmia/anosmia, rhinorrhoea, nasal obstruction, postnasal drip	Dysgeusia, Ageusia	85.6% (hyposmia/anosmia)	88.0% (dysgeusia/ageusia)	Headache
22	Vaira et al.^33^	Multicentre cohort study	May 4, 2020	345	Hyposmia/anosmia	Dysgeusia, ageusia	8.6% (isolated hyposmia/anosmia)	12.1% (isolated dysgeusia/ageusia)	None
23	Karadas et al.^34^	Prospective clinical study	June 25, 2020	239	Smell impairment	Taste impairment	18 (7.5%)	16 (6.7%)	Headache, sleep disturbance, dizziness, confusion
24	Vacchiano et al.^35^	Cross-sectional study	July 02, 2020	133	Smell disorders	Taste disorders	40 (37%)	66 (61%)	Dizziness, Headache, muscle pain
25	Cazzolla et al.^36^	Observational study	August 4, 2020	67	Olfactory disorders	Taste disorders	23 (34.3%)	50 (74.6%)	Headache (59.7%)

### Inclusion/exclusion criteria

Observational studies (cross-sectional, cohort, and case–control) with either suspected or confirmed cases of COVID-19 that reported prevalence of olfactory or gustatory dysfunction were included. This dysfunction ranged from a complete loss (anosmia/ageusia) to partial loss (hyposmia/hypogeusia) or alteration (dysosmia/dysgeusia) of these sensations. Studies in languages other than English, case reports, case series, letters to editors, and editorials were excluded.

### Quality assessment

The risk of bias in individual studies was assessed by two authors (S. A. and A. S.) using the Newcastle–Ottawa Scale (NOS). The NOS evaluates three quality parameters (selection, comparability, and outcome) divided across further items, depending on the type of study. These tables are provided as Supplementary Files: Annexures A and B.

### Statistical analysis

Statistical analyses were performed using Medcalc Statistical Software version 19.2.1 (MedCalc Software Ltd). The weighted summary proportion (prevalence) was calculated using a random-effect model because we expected to find heterogeneity across studies. Forest plots were generated to show the pooled prevalence of OGD with a 95% confidence interval (CI).

### Assessment of heterogeneity

Heterogeneity across studies was evaluated using Higgins *I*^*2*^, and a value of less than 50% for *I*^*2*^ was considered acceptable. Egger's test and a visual inspection of the funnel plot were conducted to evaluate the publication bias. A *p* value of less than 0.05 was considered significant in all cases. We intended to run a subgroup analysis in case significant heterogeneity was found between studies. The subgroup analysis would categorise studies into two groups based on the use or lack of validated instruments (validated questionnaires and chemosensory tests) to record OGD. Due to significant heterogeneity, a subgroup analysis was eventually performed for olfactory dysfunction, but sensitivity analysis had to be performed for the prevalence of gustatory dysfunction, as there was an insufficient number of studies that used validated measures to detect taste sensation.

## Results

### Study characteristics

A total of 25 studies were included in our systematic review. All the data, including study details, percentage of patients experiencing OGD, and the presence of other neurological symptoms were extracted from the studies and are presented in Table 1. The included studies' total sample size was 10,880, with individual studies’ sample sizes ranging from a minimum of 55 to a maximum of 2883. In 12 out of 25 studies, the patients also presented with other neurological problems in addition to OGD, headache being the most common. Only 17 studies that gave the exact number of patients with anosmia and hyposmia, ageusia, and dysgeusia were included in the pooled analysis (forest plot), resulting in a total sample size of 4149.

### Prevalence of gustatory dysfunction

**Figure 2:**
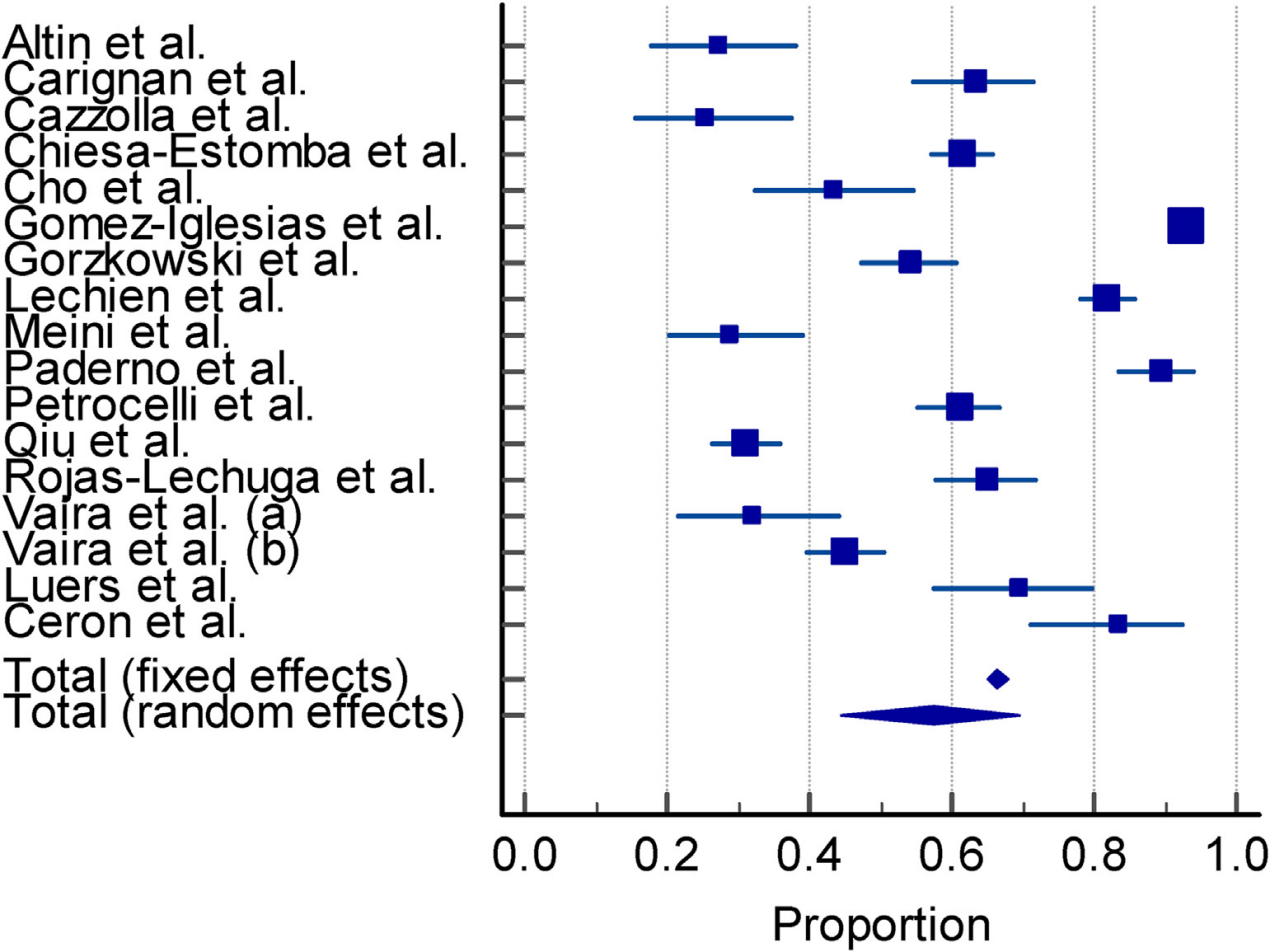
Forest plot illustrating the meta-analysis of the pooled prevalence of gustatory dysfunction in patients with COVID-19.

For evaluation of gustatory dysfunction, a total of 4149 patients included in 17 studies were identified. At least some level of gustatory dysfunction was reported in 2676 patients. Meta-analysis of the 17 included studies was performed using a random-effects model, which demonstrated a prevalence of 57.33% gustatory dysfunction among the 4149 COVID-19 patients (95% CI, 44.585%–69.600%), as shown in Figure 2. There was significant heterogeneity among the 17 studies, with an *I*^*2*^ of *p* < 0.0001 (98.50%). Visual inspection of the funnel plot (Supplementary File 1) showed an asymmetric distribution of the studies, suggesting the possibility of a publication bias. The Egger test reported a *p*-value of 0.0526.

### Prevalence of olfactory dysfunction

**Figure 3:**
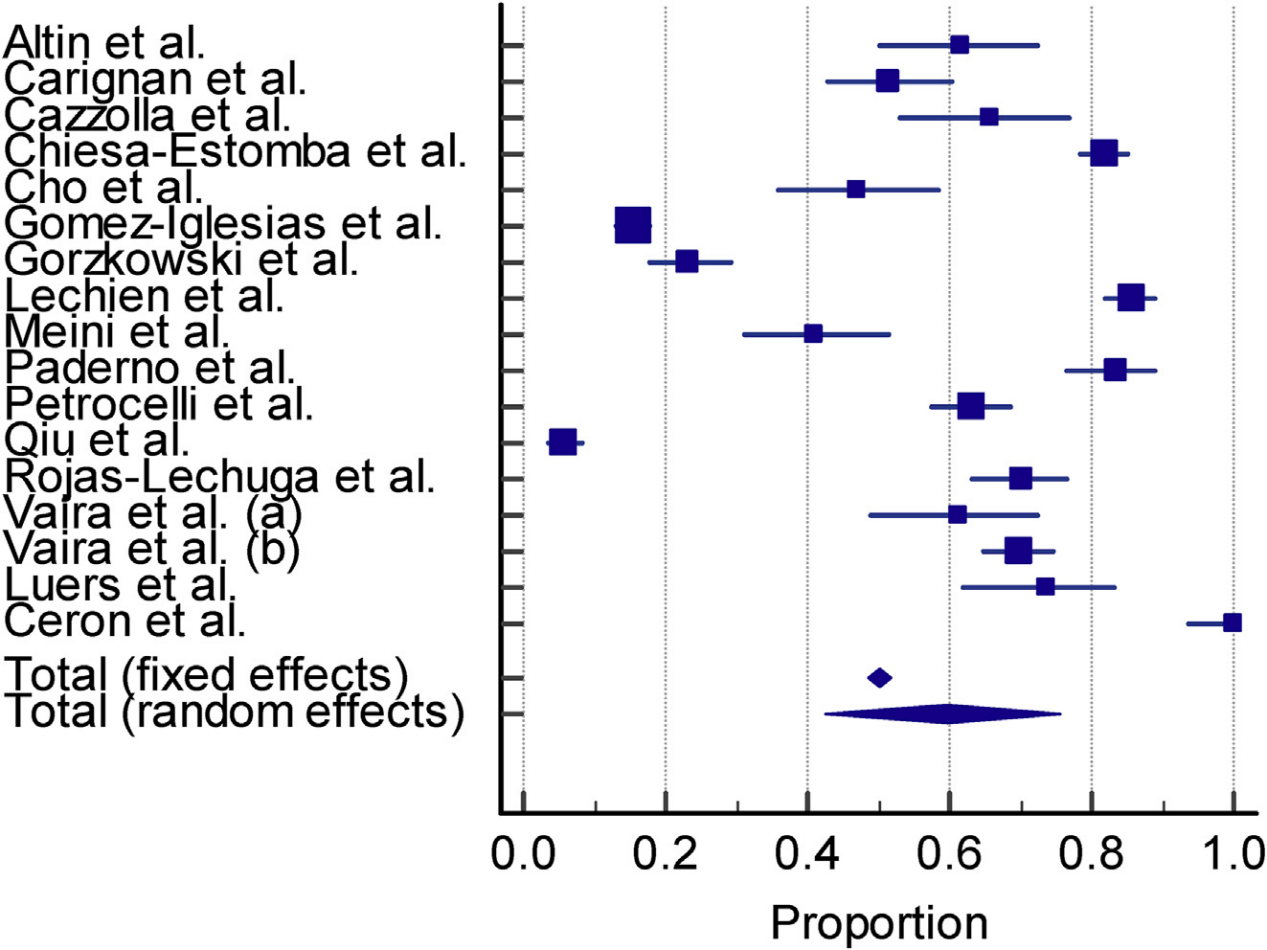
Forest plot illustrating the meta-analysis of the pooled prevalence of olfactory dysfunction in patients with COVID-19.

For evaluation of olfactory dysfunction, a total of 4149 patients included in 17 studies were identified. Some level of olfactory dysfunction was reported in 2106 total patients. Meta-analysis of the 17 included studies was performed using a random-effects model, demonstrating a prevalence of 59.69% olfactory dysfunction among the 4149 COVID-19 patients (95% CI, 42.592%–75.656%), as shown in Figure 3. There was significant heterogeneity among the studies, with an *I*^*2*^ of *p* < 0.0001 (99.17%). On visual inspection of the funnel plot (Supplementary File 2), all quadrants were seen to have a symmetric distribution, showing non-significant publication bias. The Egger test reported a *p*-value of 0.1849.

### Additional analysis

**Figure 4:**
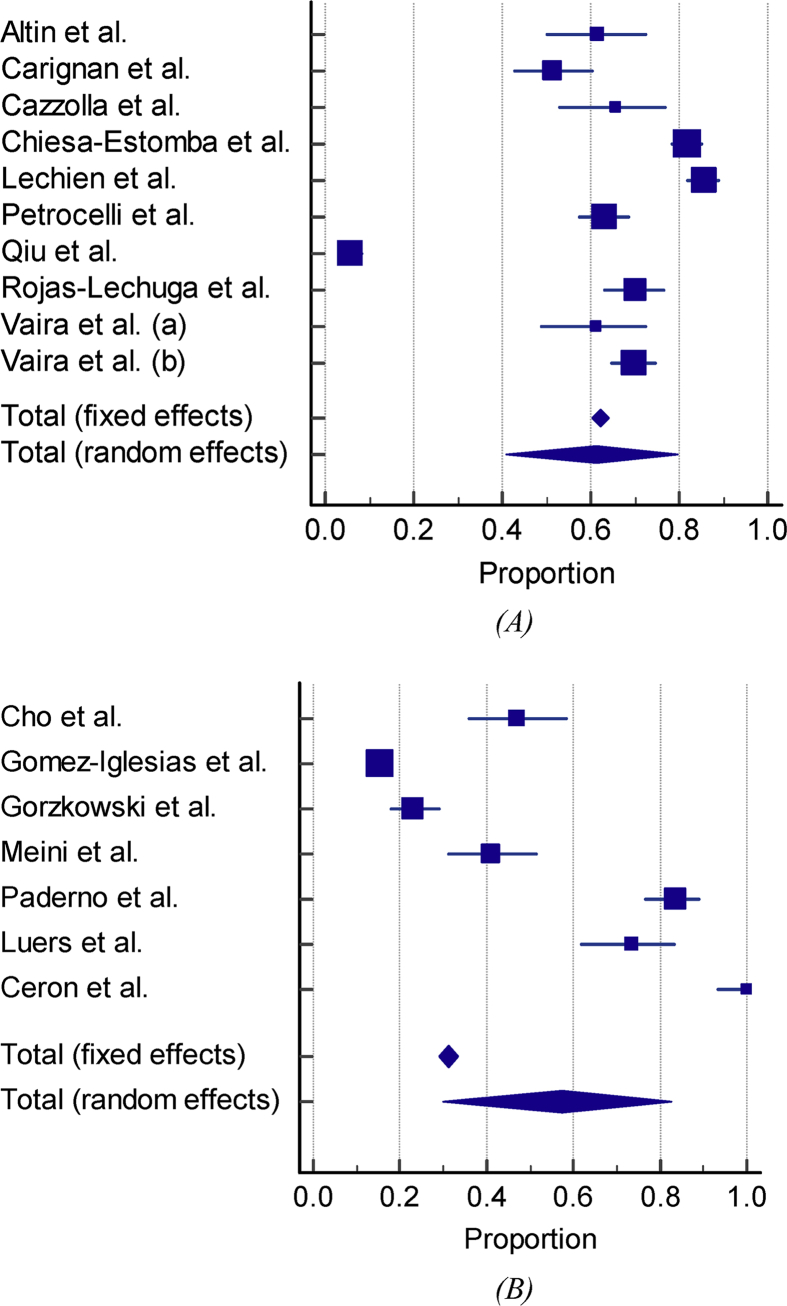
A. Subgroup analysis for the prevalence of olfactory dysfunction assessed using validated methods in patients with COVID-19. B. Subgroup analysis for the prevalence of olfactory dysfunction assessed using non-validated methods in patients with COVID-19.

**Figure 5:**
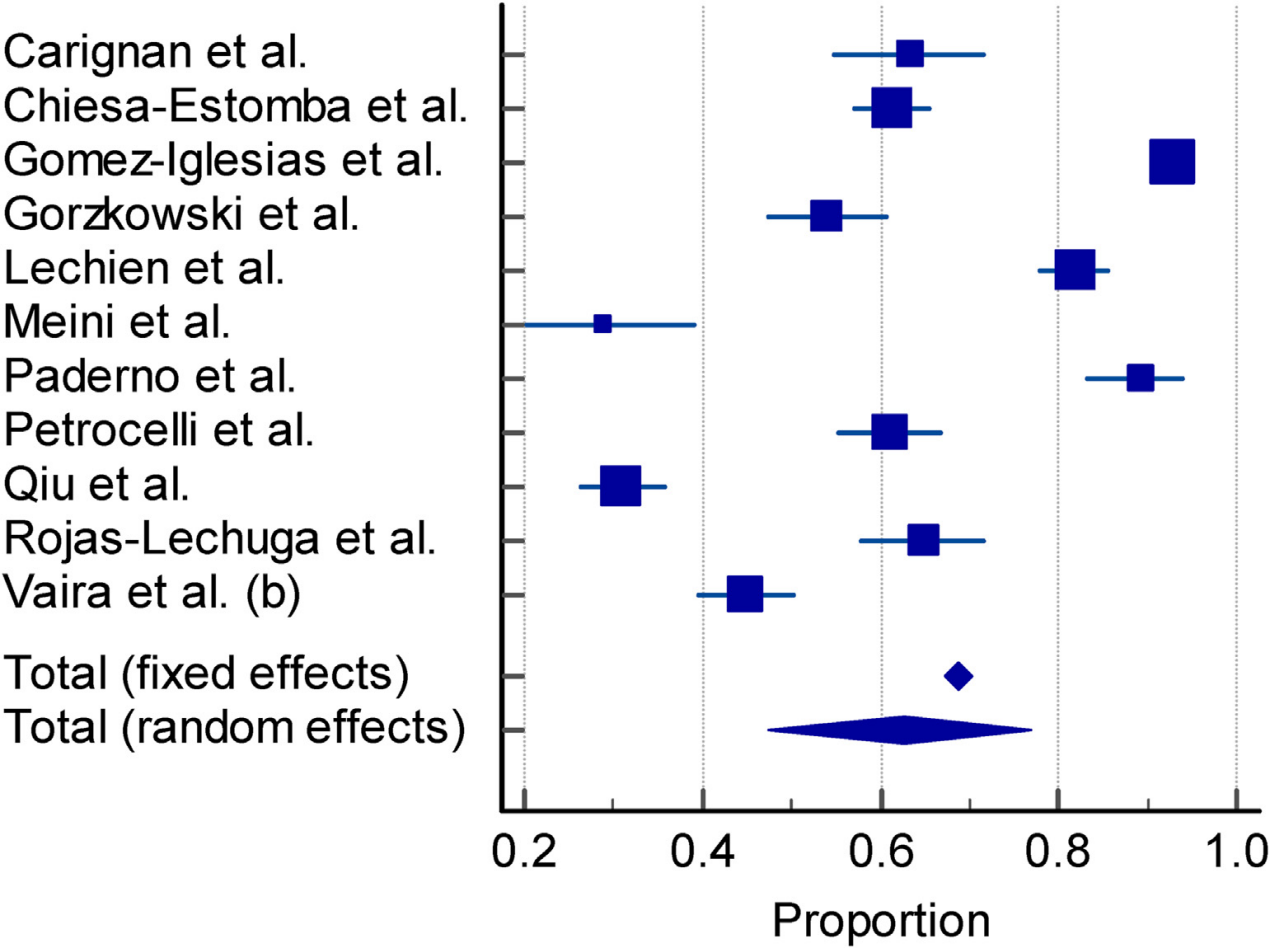
Sensitivity analysis for the prevalence of gustatory dysfunction in patients with the COVID-19 after removing studies with a small sample size (<100 participants).

Subgroup analysis (Figure 4A and B) was performed based on the method used to detect olfactory dysfunction. Studies that used validated instruments showed a pooled prevalence of 61.27% (95% CI, 40.94%–79.72%) compared to 57.43% (95% CI, 30.1%–82.49%) in studies that did not employ validated tools. Sensitivity analysis (Figure 5) for gustatory dysfunction, which was performed by removing studies with a very small sample size (<100 participants), increased pooled prevalence to 62.71% (95% CI, 47.33%–76.87%), up from the original prevalence of 57.33%.

## Discussion

Since the emergence of SARS-CoV-2 in December 2019 originating in China, it has spread globally, although the magnitude of involvement and disease severity vary across regions. The existence of this virus has been concurrent with other respiratory viruses, such as the influenza virus, making it difficult to distinguish its symptoms from other viruses that cause upper respiratory tract infection. Given the non-specific nature of the presentation, definitive diagnosis of COVID-19 mostly relies on tests like RT-PCR, although characteristic features on high-resolution computed tomography (CT) scans have also been described.^4^^,^^10^ Investigations such as CT scan and PCR are a burden on most healthcare economies; ordering them for every person presenting with non-specific symptoms like cough and fever (which may, in fact, be indicative of numerous other self-limiting/mild respiratory infections) is not economically viable. Hence, identifying clinical symptoms that distinguish COVID-19 from other closely related upper respiratory infections is key to identifying the patients that truly need these expensive investigations.^10^, ^11^, ^12^

Mao et al.^37^ initially reported anosmia in COVID-19, and since then, many researchers have started reporting new-onset OGD in COVID-19.^38^^,^^39^ The consistency in the literature led to their inclusion on the CDC's list of COVID-19 symptoms and the American Academy of Otolaryngology – Head and Neck Surgery's (AAO-HNS) publication of the Anosmia Reporting Tool for Clinicians.^40^^,^^41^ A preliminary analysis of 237 submissions to this platform reported a 73% prevalence of anosmia in COVID-19.^40^ This encouraged physicians and researchers to publish studies with evidence of OGD, and sufficient clinical data became available to exclusively attribute these symptoms to SARS-CoV-2. An earlier meta-analysis^42^ calculated the prevalence of olfactory (53%) and gustatory dysfunction (44%), but the results were limited by a small sample size. Our study included almost double the number of patients and found a higher prevalence than the previously conducted review. We believe that our results strengthen Bidkar et al.’s^43^ and Marchese et al.’s^44^ opinion that OGD should be used as a screening tool for COVID-19 and that objective chemosensory testing for these symptoms should be performed for all suspects. The use of validated tools (e.g. the COVID-19 Anosmia Reporting Tool) and chemosensory tests for detecting olfactory dysfunction showed a significantly higher prevalence previously.^42^ The subgroup analysis conducted in our study also showed an increased prevalence in the ‘validated tools group’ (61%) compared to the ‘non-validated tools group’ (57%). This implies that the true prevalence of OGD can be more reliably calculated using an objective assessment as compared to a subjective one. Sensitivity analysis run by excluding low power studies also showed a 3% increase in the prevalence of gustatory dysfunction, suggesting that the pooled prevalence calculated in this meta-analysis may, in fact, be an underestimation.

A brief review of neuroanatomy and physiology is important to understand the pathogenic mechanisms that have been reported in the literature. Cranial nerves VII, IX, and X are involved in taste perception and cranial nerve I carries olfactory sensation.^45^, ^46^, ^47^ Their peripheral parts (receptors and afferent nerves) are responsible for transmitting these sensations, while their nuclei and central connections are responsible for the relay of impulses to the brain's gustatory and olfactory centres.^48^^,^^49^ Both peripheral and central nervous systems are believed to be affected by SARS-CoV-2.^50^ Central damage is due to its neuro-invasive potential; retrograde travel through olfactory bulbs has been demonstrated in mice and is also thought to occur in humans.^51^, ^52^, ^53^, ^54^ Neurons and glial cells have also been shown to express the key receptor of this virus, i.e. the ACE-2 receptor.^55^ Lastly, SARS-CoV-2 has also been detected in cerebrospinal fluid in humans, thus strengthening the virus's neurotropic potential.^52^ On the other hand, the peripheral damage hypothesis is based on evidence that ACE-2 receptors are present on olfactory as well as glossal mucosa.^56^ Interestingly, tongue mucosa is shown to express these receptors in higher quantities compared to the adjoining oral cavity mucosa (e.g. buccal and gingival mucosa).^56^ Considering the available literature and the detailed review of the articles included in our study, the authors believe that both mechanisms might play at least some role, but evidence to suggest a predominance of one over the other is lacking. Additionally, SARS-CoV-2 binds to the sialic acid receptor. Sialic acid is an integral component of saliva that is involved in the prevention of enzymatic degradation of glycoproteins that convey gustatory molecules inside the taste pores. Hence, by binding to the sialic acid receptor, SARS-CoV-2 might increase the gustatory threshold.^57^^,^^58^

OGD may occur simultaneously without the perceived dominance of any one symptom. In fact, it is believed that taste disorders are unlikely to exist in isolation.^59^ We found a high prevalence of OGD in our study, with more than half the patients reporting some level of smell and taste dysfunction. The levels of dysfunction ranged from hyposmia/hypogeusia and dysosmia/dysgeusia to anosmia/ageusia; hence, any of the aforementioned combinations can be found. This variability may be reflective of a subjective difference rather than an objective one, since most studies did not use validated tools or objective testing to report these symptoms. Nevertheless, given such a high prevalence of at least some level of dysfunction, the authors suggest the inclusion of olfactory and gustatory manifestations as criteria for ordering expensive and time-consuming investigations like RT-PCR. This is especially important in developing countries with limited infrastructure and resources. Some scientists have also advocated the exclusive use of taste disorders as a screening tool because some level of olfactory dysfunction can be seen in other infections such as the common flu.^60^ However, we believe that isolated gustatory dysfunction is hard to find in COVID-19 patients and that a strict criterion like this will under-evaluate the burden of this disease.

Another point of clinical significance that establishes the prevalence of OGD as an important measure is its association with a better COVID-19 prognosis.^53^^,^^61^^,^^62^ Studies have reported that patients presenting with smell and taste dysfunction had less severe symptoms and were less likely to require hospitalisation through the course of their illness. Patients with OGD were less likely to need intensive care unit admission and mechanical ventilation, and death was a less frequent outcome.^63^ The pathogenesis of this association lies in the fact that patients with robust immune systems release cytokines and create a strong inflammatory response when exposed to a virus. This, in turn, leads to the degeneration/apoptosis of olfactory neurons, causing smell dysfunction. In contrast, patients who have a weak immune system are unable to create such a strong response, sparing the nasal mucosa.^64^

The authors feel the need to acknowledge some limitations encountered while deducing results from this study. First, the pooled sample size is small, and the individual studies are limited to certain geographical areas. Hence, the included studies do not reflect the OGD distribution in COVID-19 patients from every area of the world. Second, objective chemosensory testing and validated tools to detect OGD were not employed by all studies included in our review. These symptoms were described, reported, and explained as per the affected patients' subjective perception; hence, there is a high likelihood of underestimation of the true disease prevalence. Additionally, clinicians' increasing awareness of these symptoms may have led to more frequent inquiry regarding these symptoms, thus risking overestimation. Moreover, many studies were retrospective in nature; thus, the possibility of recall bias cannot be ignored. Lastly, although we performed sensitivity and subgroup analyses in our study, doing so did not significantly decrease the studies’ overall heterogeneity (I_2_). Possible reasons can be the varied geographical locations and differences in study designs and methods of detecting OGD. The strength of this meta-analysis lies in the large sample size and the recommendations/implications for clinical practice based on our results.

## Conclusion

According to our results, the prevalence of gustatory and olfactory dysfunction in COVID-19 patients was almost 57.33% and 59.69%, respectively. This dysfunction encompassed either complete or partial loss of these sensations or any noticeable alteration in them. This prevalence may, in fact, be an underestimation of the true prevalence because most included studies did not use validated tools/tests to detect OGD. The neurotropic nature of SARS-CoV-2 is explained by the high neuronal and glial cell expression of ACE-2 receptors, while direct damage to glossal and olfactory receptors plays a pathogenic role as well. Evaluating olfactory and gustatory functions both objectively and subjectively will be helpful in primary screening because PCR testing imposes significant constraints on medical resources.

## Source of funding

This research did not receive any specific grant from funding agencies in the public, commercial, or not-for-profit sectors.

## Conflict of interest

The authors have no conflicts of interest to declare.

## Ethical approval

The authors confirm that this study had been prepared in accordance with COPE roles and regulations. Given the nature of the study, IRB review was not required.

## Authors contributions

S.A. Conceptualisation, literature search, PRISMA flowchart design, data analysis, manuscript drafting and proofreading, and revision of the final version. A.S. Conceptualisation, PRISMA flowchart design, data analysis, manuscript drafting and proofreading, and revision of the final version. M.A.S.C. Literature search, data analysis and interpretation, manuscript drafting and proofreading, and revision of the final version. M.A.R. Article design refinement, manuscript drafting, data interpretation, critical revision of manuscript content, proofreading, and revision of the final version. H.F. Refining the article design, manuscript drafting, data interpretation, critical revision of manuscript content, proofreading, and revision of the final version. All authors have critically reviewed and approved the final draft and are responsible for the content and similarity index of the manuscript.
