# Supplementary material for: How common are taste and smell abnormalities in COVID-19? A systematic review and meta-analysis
Source: J Taibah Univ Med Sci. 2021 Nov 15;17(2):174–85. doi: 10.1016/j.jtumed.2021.10.009 (PMC8592522; doi:10.1016/j.jtumed.2021.10.009)
Supplement: Multimedia component 1 [file mmc1.docx]

Funnel plot assessing publication bias of gustatory dysfunction in patients with COVID-19.
