# Supplementary material for: How common are taste and smell abnormalities in COVID-19? A systematic review and meta-analysis
Source: J Taibah Univ Med Sci. 2021 Nov 15;17(2):174–85. doi: 10.1016/j.jtumed.2021.10.009 (PMC8592522; doi:10.1016/j.jtumed.2021.10.009)
Supplement: Multimedia component 3 [file mmc3.docx]

|  | **Selection** | | | | **Comparability** | | **Outcome** | | |  |
| --- | --- | --- | --- | --- | --- | --- | --- | --- | --- | --- |
| **Study** | **Representativeness of exposed cohort** | **Selection of non- exposed cohort** | **Ascertainment of exposure** | **Demonstration that outcome**  **of interest was not present at start of**  **study** | **Adjust for the most important risk factors** | **Adjust for other risk factors** | **Assessment of outcome** | **Follow-up length** | **Loss to follow-up rate** | **Total quality score** |
| Hopkins C et al. | 1 | 1 | 1 | 0 | 1 | 1 | 1 | 1 | 0 | **7** |
| Paderno et al. | 1 | 1 | 0 | 0 | 1 | 1 | 1 | 1 | 0 | **6** |
| Petrocelli et al. | 1 | 1 | 1 | 1 | 1 | 1 | 1 | 1 | 0 | **8** |
| Ceron et al. | 1 | 1 | 0 | 0 | 1 | 1 | 1 | 1 | 0 | **6** |
| Cho et al. | 1 | 1 | 1 | 0 | 1 | 1 | 1 | 1 | 0 | **7** |
| Altin F et al. | 1 | 1 | 0 | 1 | 0 | 1 | 0 | 1 | 0 | **6** |
| Karadas et al. | 0 | 1 | 1 | 1 | 1 | 1 | 1 | 1 | 0 | **7** |
| Vaira et al. | 1 | 1 | 1 | 1 | 1 | 1 | 1 | 1 | 0 | **8** |

**Annexure Table A. Detailed Newcastle-Ottawa Scale of each included cohort study**.

**Annexure Table B. Detailed Newcastle-Ottawa Scale of each included cross-sectional study.**

|  | **Selection** | | | | **Comparability** | **Outcome** | |  |
| --- | --- | --- | --- | --- | --- | --- | --- | --- |
| **Study** | **Representativeness of**  **the sample** | **Sample size** | **Non-respondents** | **Ascertainment of**  **the exposure (risk factor)** | **Confounding factors controlled** | **Assessment of outcome** | **Statistical test** | **Total quality score** |
| Lechien et al. | 1 | 1 | 1 | 1 | 1 | 1 | 1 | **7** |
| Yan et al. | 1 | 0 | 1 | 1 | 1 | 1 | 1 | **6** |
| Paderno et al. | 1 | 1 | 1 | 1 | 1 | 1 | 1 | **7** |
| Speth et al. | 1 | 0 | 1 | 1 | 1 | 1 | 1 | **6** |
| Chiesaestomba et al. | 0 | 0 | 1 | 1 | 1 | 1 | 1 | **5** |
| Rojaslechuga et al. | 0 | 1 | 1 | 1 | 1 | 1 | 1 | **6** |
| Gorzoskwi et al. | 1 | 0 | 0 | 1 | 1 | 1 | 1 | **5** |
| Luers et al. | 1 | 0 | 1 | 1 | 1 | 1 | 1 | **6** |
| Chary E et al. | 0 | 0 | 1 | 1 | 1 | 1 | 1 | **5** |
